# Identification of Key Endometrial MicroRNAs and Their Target Genes Associated With Pathogenesis of Recurrent Implantation Failure by Integrated Bioinformatics Analysis

**DOI:** 10.3389/fgene.2022.919301

**Published:** 2022-06-22

**Authors:** Jin Shang, Yan-Fei Cheng, Min Li, Hui Wang, Jin-Ning Zhang, Xin-Meng Guo, Dan-dan Cao, Yuan-Qing Yao

**Affiliations:** ^1^ Medical School of Chinese People’s Liberation Army (PLA), Beijing, China; ^2^ Shenzhen Key Laboratory of Fertility Regulation, Reproductive Medicine Center, The University of Hong Kong-Shenzhen Hospital, Shenzhen, China; ^3^ Shenzhen Institute of Advanced Technology, Chinese Academy of Science, Shenzhen, China; ^4^ Department of Obstetrics and Gynecology, The Seventh Medical Center, Chinese PLA General Hospital, Beijing, China; ^5^ Department of Obstetrics and Gynecology, The First Medical Center, Chinese PLA General Hospital, Beijing, China; ^6^ College of Medicine, Nankai University, Tianjin, China

**Keywords:** recurrent implantation failure, differentially expressed genes, differentially expressed miRNAs, bioinformatics, endometrial transcriptomics

## Abstract

**Purpose:** Recurrent implantation failure (RIF) is an enormous challenge for *in vitro* fertilization (IVF) clinicians. An understanding of the molecular mechanisms of RIF helps to predict prognosis and develop new therapeutic strategies. The study is designed to identify diagnostic biomarkers for RIF as well as the potential mechanisms underlying RIF by utilizing public databases together with experimental validation.

**Methods:** Two microarray datasets of RIF patients and the healthy control endometrium were downloaded from the Gene Expression Omnibus (GEO) database. First, differentially expressed microRNAs (miRNAs) (DEMs) were identified and their target genes were predicted. Then, we identified differentially expressed genes (DEGs) and selected hub genes through protein-protein interaction (PPI) analyses. Functional enrichment analyses of DEGs and DEMs were conducted. Furthermore, the key DEMs which targeted these hub genes were selected to obtain the key miRNA–target gene network. The key genes in the miRNA-target gene network were validated by a single-cell RNA-sequencing dataset of endometrium from GEO. Finally, we selected two miRNA–target gene pairs for further experimental validation using dual-luciferase assay and quantitative polymerase chain reaction (qPCR).

**Results:** We identified 49 DEMs between RIF patients and the fertile group and found 136,678 target genes. Then, 325 DEGs were totally used to construct the PPI network, and 33 hub genes were selected. Also, 25 DEMs targeted 16 key DEGs were obtained to establish a key miRNA–target gene network, and 16 key DEGs were validated by a single-cell RNA-sequencing dataset. Finally, the target relationship of hsa-miR-199a-5p-*PDPN* and hsa-miR-4306-*PAX2* was verified by dual-luciferase assay, and there were significant differences in the expression of those genes between the RIF and fertile group by PCR (*p* < 0.05).

**Conclusion:** We constructed miRNA–target gene regulatory networks associated with RIF which provide new insights regarding the underlying pathogenesis of RIF; hsa-miR-199a-5p-*PDPN* and hsa-miR-4306-*PAX2* could be further explored as potential biomarkers for RIF, and their detection in the endometrium could be applied in clinics to estimate the probability of successful embryo transfer.

## Introduction

Assisted reproductive technology (ART) has made a breakthrough in the history of modern science. Many infertile couples are benefited from the ART treatment, but a significant portion of them are still frustrated following multiple failed attempts, which lead to the emergence of a new challenge: recurrent implantation failure (RIF) (Coughlan et al., 2014).

RIF does not have a universal definition. The preimplantation genetic diagnosis consortium of the European Society of Human Reproduction and Embryology Preimplantation Genetic Diagnosis Consortium has defined RIF as >3 failed embryo transfers with high-quality embryos or the failed transfer of ≥10 embryos in multiple transfers, which was widely accepted (Ata et al., 2021). The etiology of RIF is complex and is not attributed to a single abnormality as a successful implantation involved a number of components (Bashiri et al., 2018). Traditionally, implantation has been considered as a process mainly involving the embryo and the endometrium, combined with other factors, such as cumulus cell competency (Benkhalifa et al., 2012). The contribution of embryo factors to RIF can be partly ruled out by performing preimplantation genetic testing for aneuploidy to select euploidy embryos for transfer (Kimelman and Pavone, 2021). However, inadequate uterine receptivity is considered to be responsible for nearly 2/3 of implantation failures (Melford et al., 2014; Craciunas et al., 2019).

The only time for embryo implantation is the window of implantation (WOI), when the endometrium enters a narrow window of a receptive state during the mid-secretory phase of the menstrual cycle (Wang W. et al., 2020). Many complex morphological and functional changes occur in the endometrial WOI during embryo implantation (von Grothusen et al., 2014). RIF is associated with molecular and functional changes of the endometrium receptivity in the WOI. Current studies of RIF patients' endometrium receptivity are often based on the transcriptomic signature, and a large number of molecules have been proposed as receptive biomarkers (Wang and Yu, 2018). However, these huge numbers of biomarkers sometimes present differences among individuals, which bring misleading judgments on the fertility status (Coutifaris et al., 2004). Therefore, it is urgent to find some credible key expressed genes in the endometrium to evaluate the endometrial receptivity. Meanwhile, microRNAs (miRNAs) have been widely reported to be involved in the function of the endometrium as transcriptional regulators of gene expression during embryo implantation (Liang et al., 2017). Studies found that the target genes of some miRNAs were involved in cyclic remodeling of the endometrium, including endometrial maturation to the receptive state, and might contribute to an adhesive interaction at the cell surface (Kang et al., 2015).

**FIGURE 1:**
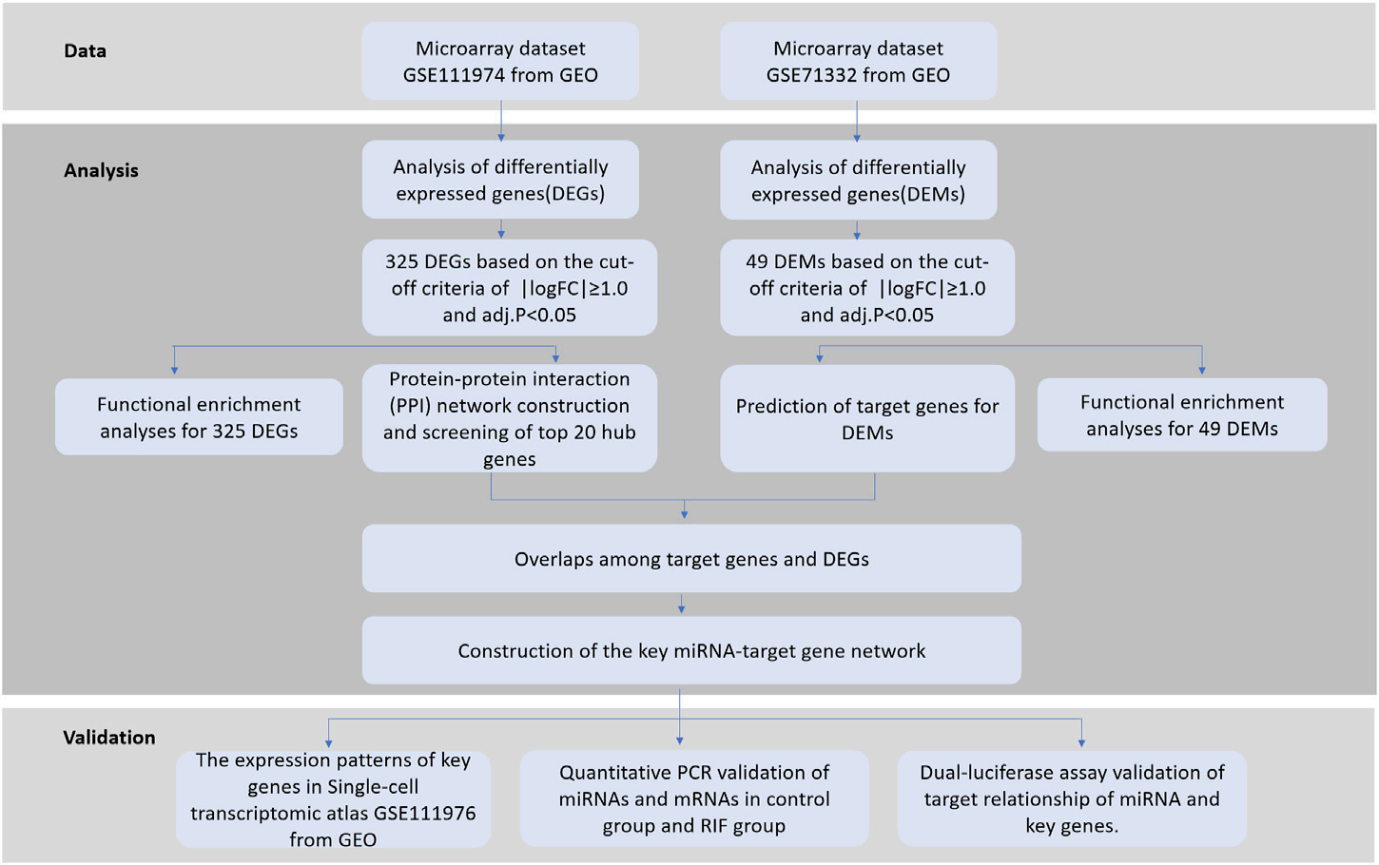
Schematic illustration of this study.

To provide insight into the potential roles of miRNAs and their target genes causing RIF, we analyzed two public microarray datasets from the Gene Expression Omnibus (GEO) database, GSE71332 (miRNA) (Shi et al., 2017) and GSE111974 (mRNA) (Bastu et al., 2019), and performed integrated bioinformatics analyses to investigate the potential endometrial causes of RIF. Differentially expressed miRNAs (DEMs) and their predicted target genes were first identified between RIF and the fertile control. Then, we identified expressed genes (DEGs) from which some key genes were obtained that constructed miRNA–target gene regulatory networks to explore the molecular mechanisms underlying RIF. The schematic illustration of this study is shown in Figure 1. Each step is elaborated in the following sub-sections.

## Methods and Materials

### Ethics Statements and Clinical Subjects

This study was approved by the Ethics Committee of the General Hospital of the People’s Liberation Army (Approval No. S2020-293-01). Participants were recruited from the First Affiliated Hospital of the People’s Liberation Army. Ten patients who failed to become pregnant after ≥ 3 embryo transfers were included in the RIF group. In the control group, six patients who conceived after their first embryo transfer or had a previous gestation history were recruited. Endometrial specimens were collected at the mid-luteal phase of a menstrual cycle, and the specimens were placed in 3 ml of the sample preservation buffer (Yikon, China) and stored at −80°C in a refrigerator until use.

### Microarray Dataset Collection

To identify the key microRNAs of RIF, we downloaded the raw data of microarray datasets using the keywords “recurrent implantation failure” combined with “microRNA” to search for genome-wide expression studies in the GEO database from the National Centre for Biotechnology Information (NCBI). The microRNA dataset GSE71332 (Shi et al., 2017) and the gene expression dataset GSE111974 (Bastu et al., 2019) were generated using the GPL18402 (Agilent-046064 Unrestricted_Human_miRNA_V19.0_Microarray) and GPL17077 (Agilent-039494 SurePrint G3 Human GE v2 8 × 60K Microarray 039381) platforms, respectively. GSE71332 contained endometrial samples collected at the WOI (LH+5 to LH+9) from seven RIF patients and five normal fertile women, while the endometrial samples collected at WOI (LH+7 to LH+10) from 24 RIF patients and 24 fertile controls were included in GSE111974. Detailed information of the two datasets is shown in Table 1.

**TABLE 1:** Characteristics of mRNA and miRNA expression profiles.

Accession	Platform	Group	Sample characteristics	Age
GSE111974	GPL17077	RIF(24)	Failure of pregnancy in ≥ 3 consecutive IVF cycles with ≥1 transfer(s) of a good quality embryo in each cycle	32.76 ± 2.33
		Control (24)	Had a history of at least one live birth with no associated comorbidities	31.13 ± 3.86
GSE71332	GPL18402	RIF(7)	Failure of implantation after three cycles of IVF or ICSI with no less than 10 good quality embryos	31.57 ± 4.50
		Control (5)	Got pregnancy after embryo implantation no more than three times	31 ± 3.16

The first dataset is mRNA, expression profiles, and the second one is miRNA, expression profiles.

### Differential Gene Expression Analysis

Series Matrix Files of GSE71332 were downloaded and imported into the R-studio (Version: 1.2.1335), and raw data of GSE111974 were downloaded from the GEO into R-studio as well. The robust multi-array average (RMA) method was used for data preprocessing (Bolstad et al., 2003; Irizarry et al., 2003). The non-human gene probes from the chips were removed, and multiple probes related to the same gene were reduced to one and summarized as median values. mRNA microarray probes were annotated using the annotated files in the platform GPL18402 while removing probes that did not match any gene symbols.

DEMs and DEGs were identified by the R Bioconductor package “limma” (Version: 3.46.0) (Ritchie et al., 2015) by comparing the RIF and healthy control groups. For DEM and DEG identification, | log2FC| > 1 and adjusted *p* < 0.05 were regarded as statistically significant.

### Functional and Pathway Enrichment Analysis of Differentially Expressed miRNAs and Differentially Expressed Genes

The Gene Ontology (GO) terms of biological processes, cellular components, and molecular functions were used to determine the function of the DEMs and DEGs. Kyoto Encyclopedia of Genes and Genomes (KEGG) pathway analysis was used to explore the significant pathways of DEGs. We performed GO enrichment analysis for DEMs using an open access, standalone functional enrichment tool, FunRich (http://www.funrich.org) (Pathan et al., 2015). For DEGs, we used the R package clusterProfiler package (version: 3.18.1) (Yu et al., 2012), and KEGG pathway analyses were performed using the Cytoscape (version: 3.8.2) with the ClueGo (version: 2.5.7) and the CluePedia (version: 1.5.7) plugin. The *p*-values were all less than 0.05, which were calculated using a two-sided hypergeometric test and Benjamini–Hochberg adjustment.

### Protein-Protein Interactions: Network Construction and Selection of Hub Genes

Protein-protein interactions (PPIs) of DEGs were screened using the STRING database (version: 11.0) (Szklarczyk et al., 2019), with the confidence threshold value ≥ 0.4 (medium confidence). Networks were visualized using the Cytoscape (version: 3.8.2, https://www.cytoscape.org/). The CytoNCA plugin (version: 2.1. 6) (Tang et al., 2015) was used to analyze network topology properties for nodes, and the score of gene nodes was calculated using three centrality methods (degree centrality, betweenness centrality, and closeness centrality) (Cukierski and Foran, 2008; Opsahl et al., 2010; Du et al., 2015). In addition, a Cytoscape-plugin Molecular Complex Detection (MCODE) (Bader and Hogue, 2003) (version: 2.0.0, http://apps.cytoscape.org/apps/MCODE) was adopted to select the key modules of the PPI network with degree cutoff = 2, max. depth = 100, node score cutoff = 0.2, and k-Core = 2. Several top nodes with high degrees based on the three centrality methods and key modules of the PPI network were identified as hub genes for further analysis.

### Integrative Analysis of Differentially Expressed miRNAs and Differentially Expressed Genes

We predicted the potential target genes of the DEMs from GSE71332 using miRWalk (Sticht et al., 2018) (version: 2.0, https://mirwalk.umm.uni-heidelberg.de), an miRNA–mRNA binding site prediction tool (Ding et al., 2016). Based on the typical inhibitory effect of a miRNA on its downstream target, only upregulated miRNA–downregulated mRNA and downregulated miRNA–upregulated mRNA pairs were identified by checking the direction of expression change of predicted miRNA–target pairs in DEMs and DEGs. The regulatory network of the identified DEM–DEG pairs was constructed and visualized using the Cytoscape (version: 3.8.2). Moreover, the network topology was analyzed to identify key miRNA–target gene pairs.

### Expression Analysis of Key Genes in the Single-Cell RNA-Sequencing Dataset of Endometrium Samples

To identify the key genes demonstrating variation during the whole menstrual cycle between control and RIF samples, the raw data of a single-cell RNA-seq transcriptome profile, GSE111976 (Wang W. et al., 2020), was downloaded from the NCBI GEO database. The GSE111976 dataset covers the single-cell transcriptome profile of endometrium biopsies sampled from 19 healthy and fertile females at 4–27 days of their menstrual cycle, which can be used to systematically characterize endometrial transformation across the healthy human menstrual cycle in preparation for embryo implantation. We identified the expression of the key genes at the mid- and late-secretory stages in six cell types: stromal fibroblasts, unciliated endothelial cells, ciliated endothelial cells, macrophages, and lymphocytes. The expression of key genes was analyzed in the four stages.

### Verification of Selected Key Gene Expression and its Upstream miRNA in Clinical Samples

Total RNA was extracted from endometrial cells using an RNA Quick Purification kit (ES Science, China). The determination of RNA concentration and purity was performed using a spectrophotometer (Nanodrop 2000, Thermo Scientific). Total RNA with good purity and integrity was reverse-transcribed using a Fast All-in-One RT Kit (ES Science, China) and a Mir-X miRNA First-Strand Synthesis kit (Takara, Japan), respectively. Then, cDNA samples were stored at −80°C. Real-time quantitative polymerase chain reaction (qPCR) was performed to determine the key miRNA–target gene pair expression levels*.* miRNA expression was measured using a SYBR®Green kit (Takara, Japan) with an ABI StepOne PCR instrument. *U6* was used as an internal control for miRNA, and *GAPDH* was used as an internal control for mRNA. Relative quantification of gene expression was performed using the comparative CT (2^−△△CT^) method.

### Validation of miRNA–Target Relationship by Dual-Luciferase Reporter Assay

The 3′-untranslated regions (UTRs) of the target genes were amplified by PCR using the genomic DNA of 293T cells as a template and the designed amplification primers (Table 2). Both wild-type and mutated forms of the UTRs for the genes were synthesized. After cloning the amplified WT3′UTR (wild-type vector) or MUT 3′UTR (mutant vector) into the pmir-GLO vector, validation of the reporter assay was performed with the dual luciferase reporter system (Promega E1910, United States). For detection of relative fluorescence values, an miRNA mimic or non-target control was respectively co-transfected into 293T cells withWT3′UTR pmir-GLO-Reporter or MUT 3′UTR pmir-GLO-Reporter. The luciferase fluorescence was measured using a SpectraMax®i3 (Molecular Devices, United States).

**TABLE 2:** Dual-luciferase assay primer.

PDPN-WT-pmirGLO	Cac​ttg​cct​ggc​cca​ctc​aga​atc​cac​ggt​gac​ctc​tcc​gct​tgc​caa​aat​aac​cga​agg​aaa​gac​cgt​tca​cca​gac​ttg​gct​cct​cta​aac​att​tgc​tgt​tca​aac​atg​ttt​ttg​aat​ata​cat​tct​ata​aaa​gat​tat​ttg​aaa​gac​aaa​att​cat​aga​aaa​tgg​agc​aaa​act​gta​taa​act​gat​ttg​taa​cta​aca​ctg​gac​cat​tgg​atc​gat​att​ata​tgc​tgt​aac​cat​gtg​tct​ccg​tct​gac​cat​tct​tgt​tat​tgt​taa​aat​gca​gag​gaa​tct​gga​aat​att​tat​atc​cac​gga​gtc​ctt​gga​tcc​agt​gct​acg​tca​gta​aat​agc​acc​agc​att​ttg​caa​ttg​ctg​atc​tgc​tga​aat​gta​cac​att​ctg​gtc​tag​ttt​ggt​ct
PDPN-MUT-pmirGLO	Cac​ttg​cct​ggc​cca​ctc​aga​atc​cac​ggt​gac​ctc​tcc​gct​tgc​caa​aat​aac​cga​agg​aaa​gac​cgt​tca​cca​gac​ttg​gct​cct​cta​aac​att​tgc​tgt​tca​aac​atg​ttt​ttg​aat​ata​cat​tct​ata​aaa​gat​tat​ttg​aaa​gac​aaa​att​cat​aga​aaa​tgg​agc​aaa​act​gta​taa​act​gat​ttg​taa​cta​tgt​gac​ctc​cat​tgg​atc​gat​att​ata​tgc​tgt​aac​cat​gtg​tct​ccg​tct​gac​cat​tct​tgt​tat​tgt​taa​aat​gca​gag​gaa​tct​gga​aat​att​tat​atc​cac​gga​gtc​ctt​gga​tcc​agt​gct​acg​tca​gta​aat​agc​acc​agc​att​ttg​caa​ttg​ctg​atc​tgc​tga​aat​gta​cac​att​ctg​gtc​tag​ttt​ggt​ct
PAX2-WT-pmirGLO	gcc​ccg​ggc​ggc​cga​agg​ccg​ggc​cgc​ccc​gtc​ccg​ccc​cgt​agt​tgc​tct​ttc​ggt​agt​ggc​gat​gcg​ccc​tgc​atg​tct​cct​cac​ccg​tgg​atc​gtg​acg​act​cga​aat​aac​aga​aac​aaa​gtc​aat​aaa​gtg​aaa​ata​aat​aaa​aat​cct​tga​aca​aat​ccg​aaa​agg​ctt​gga​gtc​ctc​gcc​cag​atc​tct​ctc​ccc​tgc​gag​ccc​ttt​tta​ttt​gag​aag​gaa​aaa​gag​aaa​aga​gaa​tcg​ttt​aag​gga​acc​cgg​cgc​cca​gcc​agg​ctc​cag​tgg​ccc​gaa​cgg​ggc​ggc​gag​ggc​ggc​gag​ggc​gcc​gag​gtc​cgg​ccc​atc​cca​gtc​ctg​tgg​ggc​tgg​ccg​ggc​aga​gac​ccc​gga​ccc​agg​ccc​agg​cct​aac​ctg​cta​aat​gtc​ccc​gga​cgg
PAX2-MUT-pmirGLO	Gcc​ccg​ggc​ggc​cga​agg​ccg​ggc​cgc​ccc​gtc​ccg​ccc​cgt​agt​tgc​tct​ttc​ggt​agt​ggc​gat​gcg​ccc​tgc​atg​tct​cct​cac​ccg​tgg​atc​gtg​acg​act​cga​aat​aac​aga​aac​aaa​gtc​aat​aaa​gtg​aaa​ata​aat​aaa​aat​cct​tga​aca​aat​ccg​aaa​agg​ctt​gga​gtc​ctc​gcc​cag​atc​aga​gag​ggc​tgc​gag​ccc​ttt​tta​ttt​gag​aag​gaa​aaa​gag​aaa​aga​gaa​tcg​ttt​aag​gga​acc​cgg​cgc​cca​gcc​agg​ctc​cag​tgg​ccc​gaa​cgg​ggc​ggc​gag​ggc​ggc​gag​ggc​gcc​gag​gtc​cgg​ccc​atc​cca​gtc​ctg​tgg​ggc​tgg​ccg​ggc​aga​gac​ccc​gga​ccc​agg​ccc​agg​cct​aac​ctg​cta​aat​gtc​ccc​gga​cgg

### Statistical Analysis

We used the GraphPad Prism software (version: 8.0.1) for statistical analysis. Data are expressed as mean ± standard error. Differences were analyzed using Student’s t-test. Statistical significance was set at *p* < 0.05.

## Results

### Identification of Differentially Expressed miRNAs in Recurrent Implantation Failure

**FIGURE 2:**
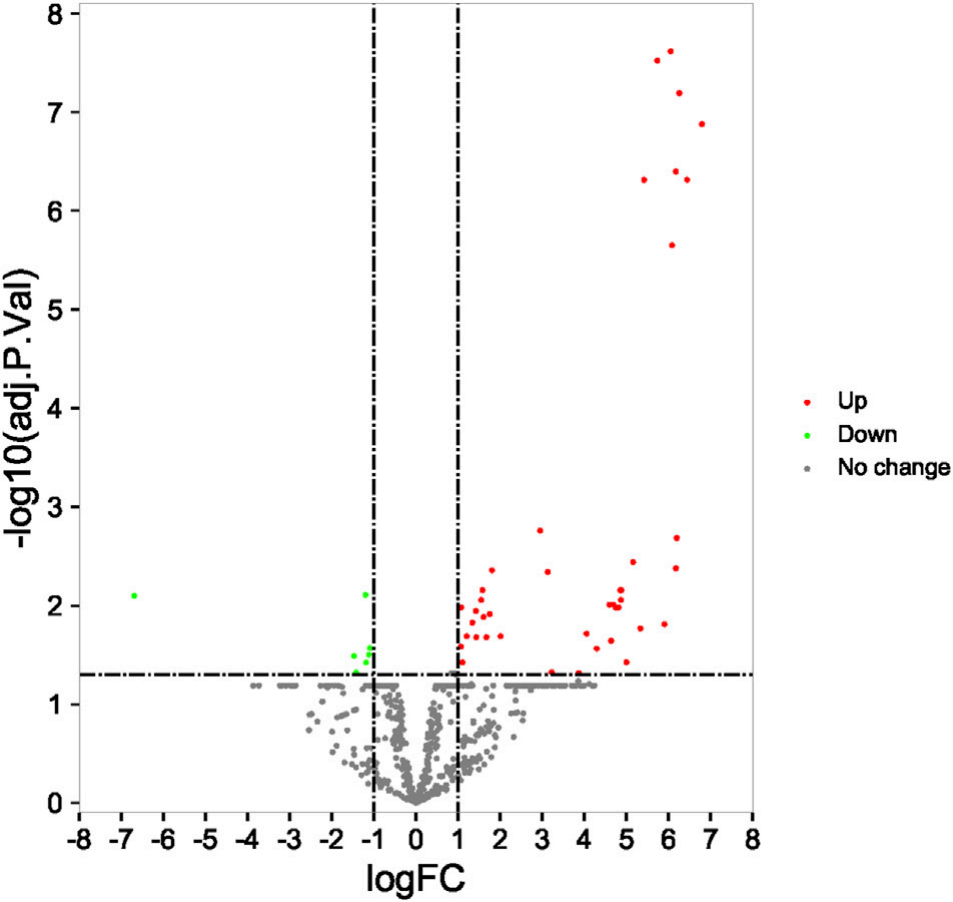
Identification of differentially expressed miRNA in profile datasets. The 49 differentially expressed miRNAs in GSE71332.

A total of 49 DEMs (42 upregulated and seven downregulated) were identified between the RIF and control groups, and volcano plots with their corresponding log2FC values are shown in Figure 2.

### Predicting Target Genes of Differentially Expressed miRNAs and Functional Enrichment Analysis

The online tool of miRWalk (Version: 2.0) based on 6 bioinformatic was utilized to predict the potential candidate target genes of aberrant miRNAs, such as the algorithms miRWalk 2.0, Pictar 2, PITA, RNA22v2, RNAhybrid 2.1, and TargetScan 6.2. Only the target genes which were common in the prediction of all the aforementioned algorithms were screened out. Finally, a total of 136,678 target genes for 49 DEMs were obtained.

**FIGURE 3:**
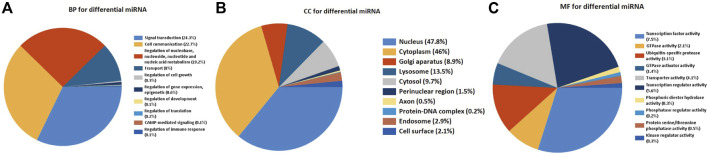
Functionally enriched GO term analysis of DEMs in RIF. BP-associated category, CC-associated category, and MF-associated category of DEMs. DEMs, differentially expressed miRNAs; GO, Gene Ontology; BP, biological processes; CC, cellular component; MF, molecular function.

In order to get insight into the functions and mechanisms of these DEMs, the biological process (BP), cellular component (CC), and molecular function (MF) were enriched in GO terms. The most significantly enriched GO terms in the BP-associated category was signal transduction. In the CC category, 49 miRNAs were suggested to be related to the nucleus and cytoplasm. In the MF category, DEMs were mainly involved in transcription factor activity (Figures 3A–C).

### Identification of Recurrent Implantation Failure Differentially Expressed Genes and Functional Enrichment Analysis

**FIGURE 4:**
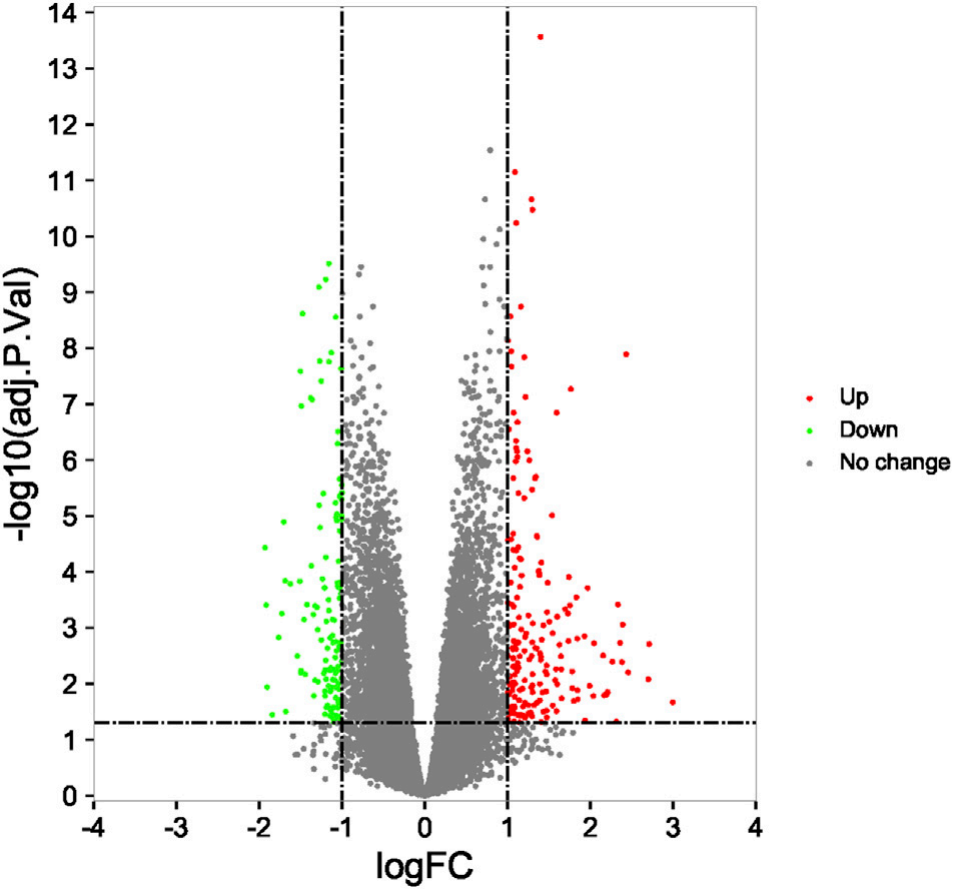
Identification of differentially expressed genes and enrichment analysis. The 325 differentially expressed mRNAs in GSE111974.

Compared with the RIF patients and fertile control groups in GSE111974 datasets by the R Bioconductor package ‘limma’ (version: 4.0.3), 325 DEGs (200 upregulated and 125 downregulated) were identified for further analysis (Figure 4).

**FIGURE 5:**
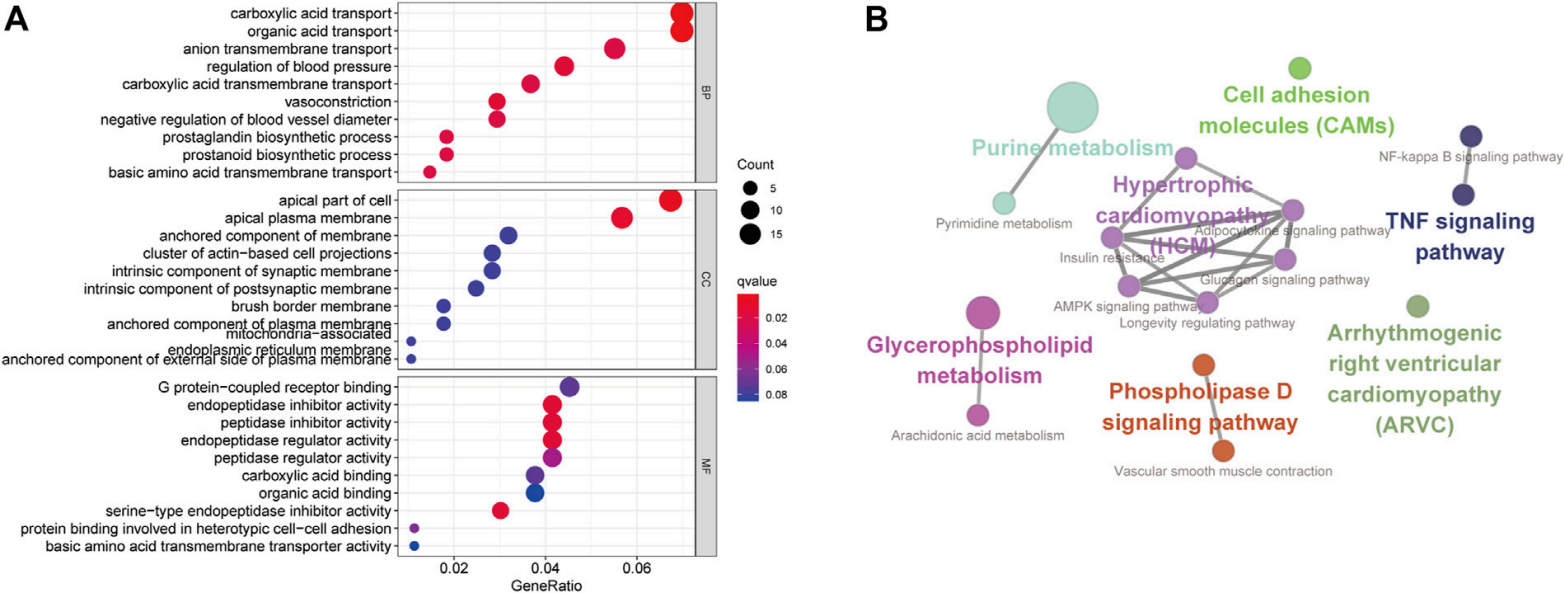
Functionally enriched GO terms and KEGG pathway analysis of DEGs in RIF. **(A)** BP-associated category, CC-associated category, and MF-associated category of DEMs; DEGs, differentially expressed genes; GO, Gene Ontology; **(B)** KEGG pathways; KEGG, Kyoto Encyclopedia of Genes and Genomes.

In order to have an in-depth understanding of biological significance of these DEGs, we performed GO enrichment by the R package clusterProfiler package and KEGG enrichment using the Cytoscape software with ClueGo and CluePedia plugin (Figure 5A). In GO enrichment analysis, the BP-associated category was significantly enriched in carboxylic acid transport and organic acid transport. The identified DEGs were significantly enriched in the apical part of the cell and the apical plasma membrane in the CC category. However, in the MF category, the DEGs were mainly enriched in endopeptidase inhibitor activity. The most significantly enriched KEGG terms of the DEGs included arrhythmogenic right ventricular cardiomyopathy (ARVC) (*p* = 0.03), TNF signaling pathway (*p* = 0.03), vascular smooth muscle contraction, cell adhesion molecules (CAMs) (*p* = 0.03), purine metabolism, pyrimidine metabolism (*p* = 0.001), NF-kappa B signaling pathway (*p* = 0.02), arachidonic acid metabolism (*p* = 0.02), phospholipase D signaling pathway (*p* = 0.01), AMPK signaling pathway (*p* = 0.04), longevity regulating pathway (*p* = 0.01), adipocytokine signaling pathway (*p* = 0.02), glucagon signaling pathway, and insulin resistance (*p* = 0.03) (Figure 5B).

### Differentially Expressed Gene Prioritization by Protein-Protein Interaction Network Analysis and Key Target Gene Selection

**FIGURE 6:**
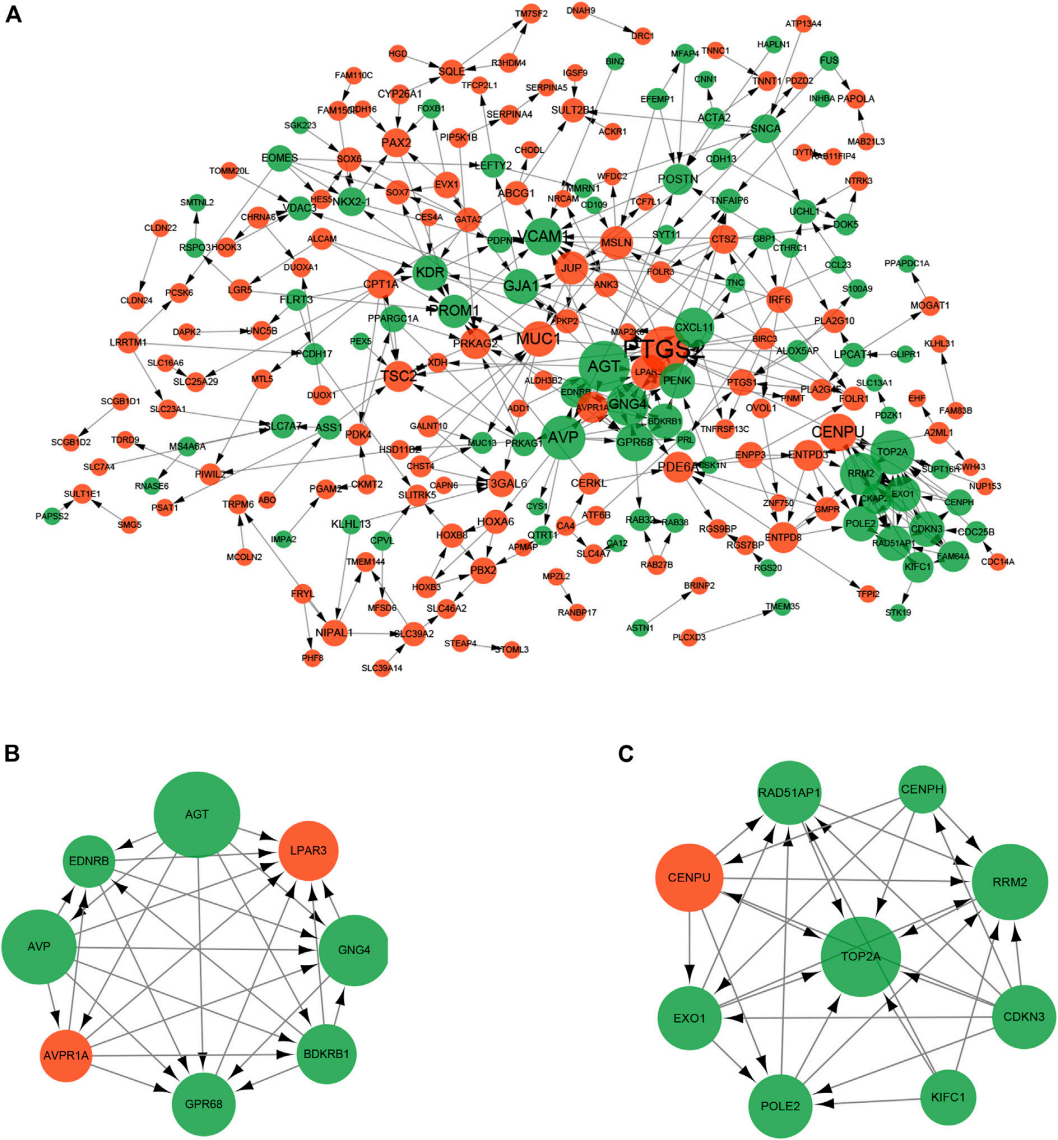
Protein-protein interaction (PPI) network of DEGs. The size of the dot represents the degree of association of the node genes. The larger the dot, the greater the degree of genetic association. Red nodes and green nodes indicate upregulated and downregulated genes, respectively. **(A)** PPI network contained 229 nodes and 373 interaction pairs; **(B)** Sub-network module with the highest score in the PPI network; **(C)** Sub-network module with the second highest score in the PPI network.

To investigate the interactions of DEGs, we constructed the PPI network for the 325 DEGs. The PPI network contained 229 nodes and 373 interaction pairs (Figure 6A). A total of 11 key cluster modules were screened out by the MCODE plugin. Figures 6B,C show the first two key modules of the PPI network; one contained eight nodes and 28 interaction pairs, and the other contained nine nodes and 30 interaction pairs. CytoNCA was used for the network topology analysis based on the three centrality methods (degree centrality, betweenness centrality, and closeness centrality). Table 3 shows the top 20 nodes with high degrees of network topology analysis. Then 11 upregulated genes and 22 downregulated genes were screened as hub genes, such as lysophosphatidic acid receptor 3 (*LPAR3*), angiotensinogen (*AGT*), and podoplanin (*PDPN*).

**TABLE 3:** Top 20 nodes of DEGs in the PPI network conducted based on 325 DEGs.

Gene	Degree	Gene	Betweenness	Gene	Closeness
AGT	16	PTGS2	14450.87	PTGS2	0.038592
PTGS2	14	MUC1	6803.066	AGT	0.038429
TOP2A	13	AGT	6584.209	VCAM1	0.038229
AVP	13	VCAM1	6000.786	GJA1	0.038185
RRM2	12	CENPU	5574.618	GPR68	0.038057
GNG4	12	AVP	5513.234	MUC1	0.038044
CXCL11	11	GJA1	5251.475	KDR	0.037968
CENPU	10	TSC2	5165.327	PROM1	0.037924
GPR68	10	PROM1	5011.375	GNG4	0.037918
VCAM1	10	KDR	4198.465	AVP	0.03778
MUC1	9	GNG4	4130.481	TSC2	0.037755
EXO1	9	PDE6A	3660.51	JUP	0.037673
RAD51AP1	9	ST3GAL6	3071.586	PRKAG2	0.037661
POLE2	9	POSTN	2746.785	CXCL11	0.037661
CDKN3	9	PAX2	2703.252	CENPU	0.037611
KDR	9	ASS1	2523.089	LPAR3	0.037611
LPAR3	9	PRKAG2	2443.258	BDKRB1	0.037611
PENK	9	ABCG1	2398.07	PDPN	0.037599
GJA1	9	NKX2-1	2393.507	PENK	0.037574
BDKRB1	9	KLHL13	2329.919	POSTN	0.037556

Degree: results of the degree centrality algorithm; betweenness: results of the betweenness centrality algorithm; closeness: results of the closeness centrality algorithm.

**FIGURE 7:**
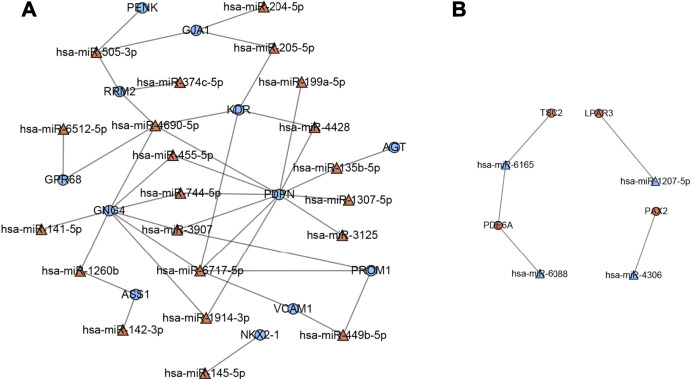
Key miRNA-mRNA network. Triangles represent DEMs, and circles represent target DEGs. **(A)** Blue circles indicate downregulated genes, and red triangles indicate upregulated miRNAs. **(B)** Red circles indicate upregulated genes, and blue triangles indicate downregulated miRNAs.

A total of 16 hub genes from PPI network analysis were identified, which had predicted target relationship with these DEMs (Figures 7A,B). As a result, we regarded these 16 genes (*LPAR3, PAX2, PDE6A, TSC2, AGT, ASS, GJA1, GNG4, GPR68, KDR, NKX2-1, PDPN, PENK, PROM1, RRM2,* and *VCAM1*), including four upregulated and 12 downregulated, as key genes in our analysis (Table 4).

**TABLE 4:** Key genes and miRNAs in the network.

	Genes	MiRNAs
Upregulated	LPAR3 \ PAX2 \ PDE6A \ TSC2	hsa-miR-744-5p \ hsa-miR-6717-5p \ hsa-miR-6512-5p \ hsa-miR-505-3p \ hsa-miR-4690-5p \ hsa-miR-455-5p \ hsa-miR-449b-5p \ hsa-miR-4428 \ hsa-miR-3907 \ hsa-miR-374c-5p \ hsa-miR-3125 \ hsa-miR-205-5p \ hsa-miR-204-5p \ hsa-miR-199a-5p \ hsa-miR-1914-3p \ hsa-miR-145-5p \ hsa-miR-142-3p \ hsa-miR-135b-5p \ hsa-miR-1307-5p \ hsa-miR-1260b
Downregulated	AGT \ ASS1 \ GJA1 \ GNG4 \ GPR68 \ KDR \ NKX2-1 \ PDPN \ PENK \ PROM1 \ RRM2 \ VCAM1	hsa-miR-1207-5p \ hsa-miR-4306 \ hsa-miR-6088 \ hsa-miR-6165

### Single-Cell RNA-Sequencing Dataset Analysis of the Prioritized Target Genes

**FIGURE 8:**
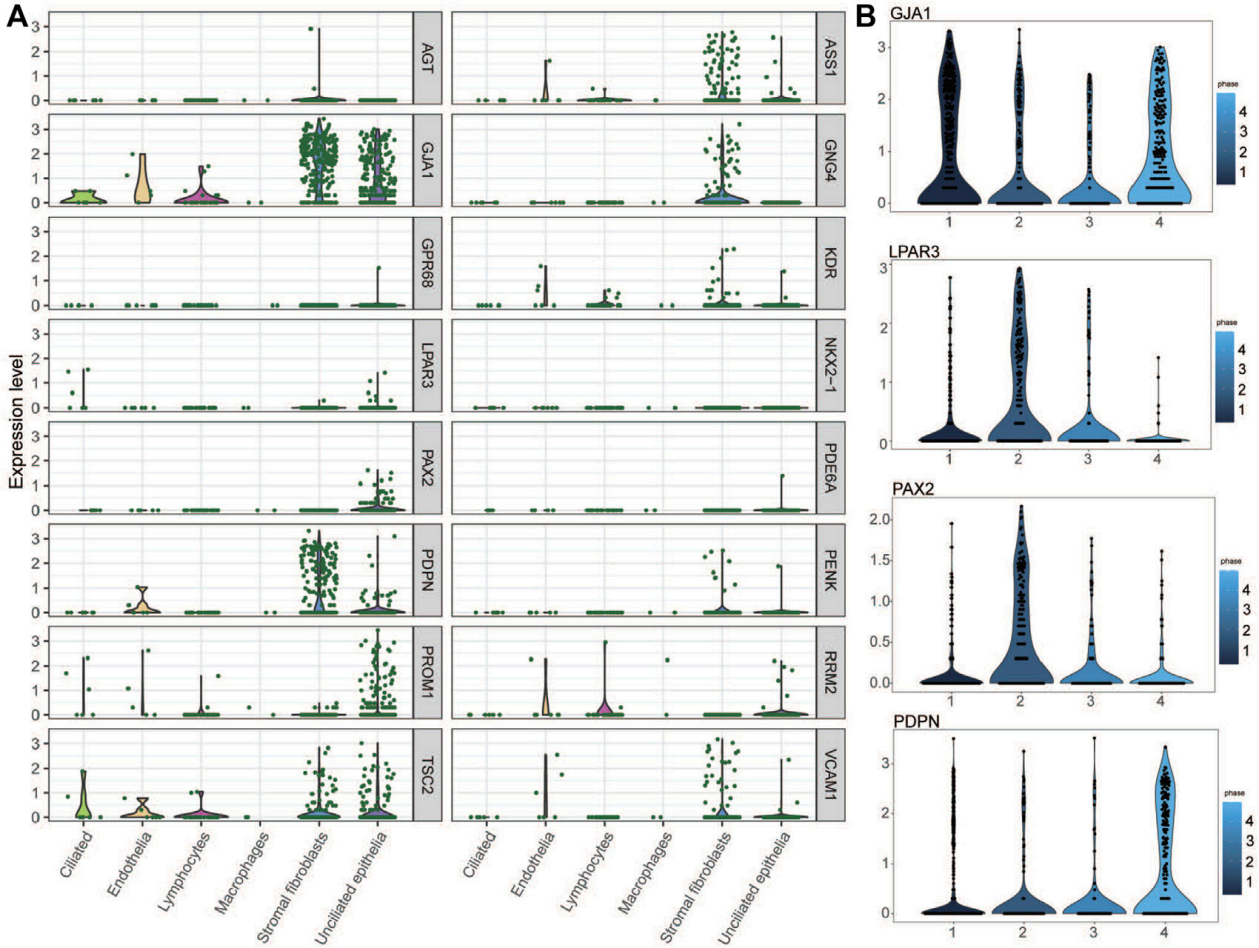
Key genes in the single-cell RNA-seq transcriptome profile of the normal endometrium. **(A)** Expression of key genes in the mid- and late-secretary menstrual phase. **(B)** *PDPN, GJA1, LPAR3,* and *PAX2* expression variation during four menstrual phases.

The expression levels of the key genes in the normal endometrium were examined in a single-cell sequencing dataset, which divides the normal female menstrual cycle into four phases (menstrual and early-proliferative phase, late-proliferative phases, early-secretory phase, and mid- and late-secretory phase). As shown in Figure 8A, most of the key genes were distinctly expressed in stromal fibroblasts and unciliated epithelial cells during the mid- and late-secretory phase. We believe that the changes in endometrial gene expression during WOI may be the key period for embryo implantation. Therefore, we found genes (*PDPN*, *GJA1*, *LPAR3*, and *PAX2*) with significant expression changes during WOI from the single-cell data set of the normal endometrium (Figure 8B). *PDPN* and *GJA1* exhibited upregulation when entering the mid-secretory stage, whereas the expression of *LPAR3* and *PAX2* gradually decreased from the late-proliferative phases to the mid- and late-secretory phases. Interestingly in GSE111974, the expressions of *PDPN* and *GJA1* were downregulated in RIF patients in the window of receptivity of the endometrium when compared with that of healthy people, whereas those of *LPAR3* and *PAX2* were upregulated in RIF patients. In the mid-secretory phase, the endometrium prepares for embryo attachment. Therefore, these results indicated that dysregulation of *PDPN, GJA1, LPAR3,* and *PAX2* expression in RIF patients might disturb endometrial receptivity and affect embryo implantation.

### Experimental Validation of the Significant miRNA–Target Gene Pairs

**FIGURE 9:**
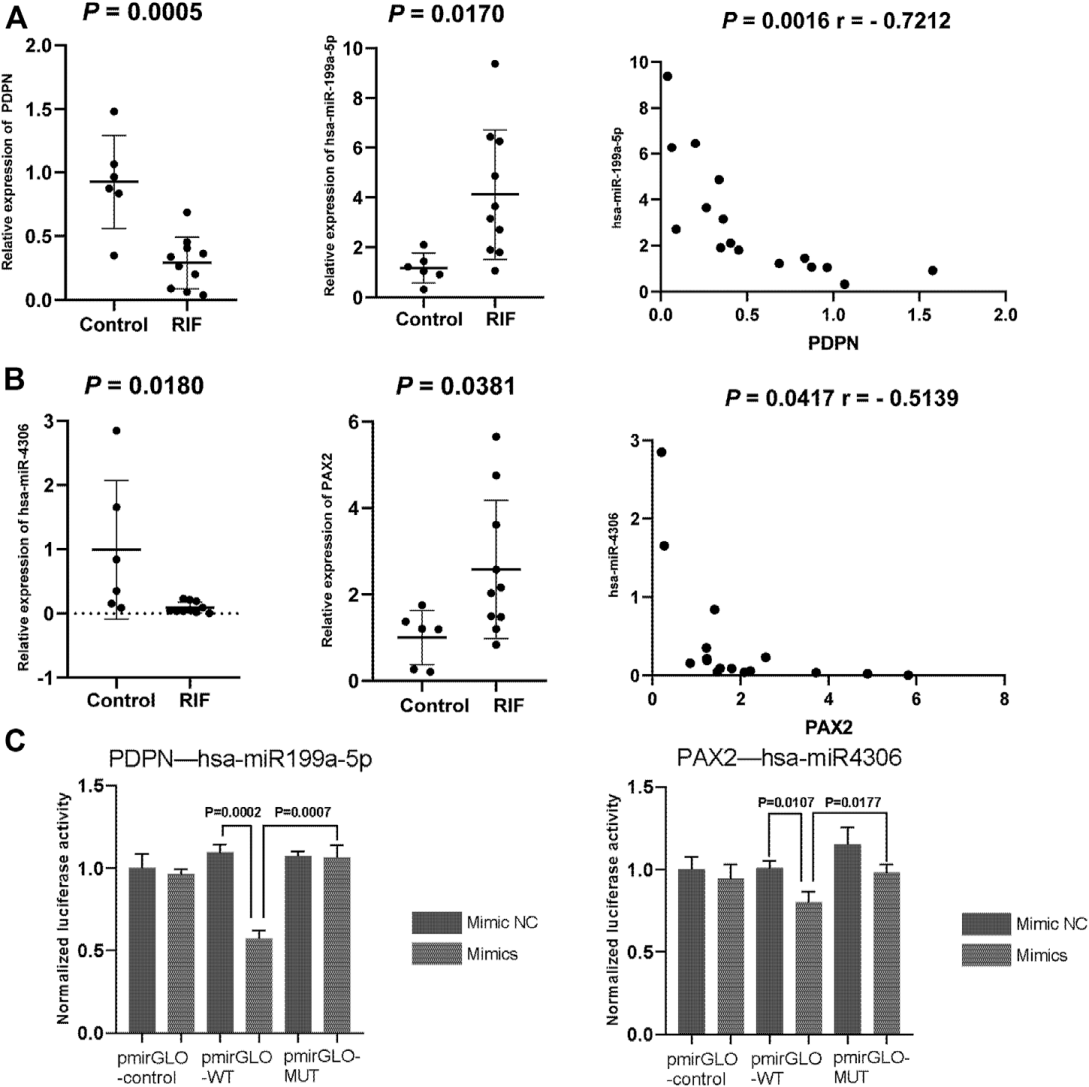
**(A,B)** Quantitative PCR validation of miRNAs and mRNAs in the control group and RIF group. The expression of **(A)** *PDPN* and hsa-miR-199a-5p and **(B)** *PAX2* and hsa-miR-4306. **(C)** Dual-luciferase assay validation of target relationship of miRNA and genes. Data are presented as the normalized fold change. Experiments were repeated in triplicate, and values are expressed as mean ± SEM. * tive. A; miRNA or miR, microRNA.

Two miRNA–target gene pairs were selected for further experimental validation due to their function in the uterine or adnexa in existing reports that were related to RIF. The expression and regulatory relationships of hsa-miR-4306 with *PAX2* and hsa-miR-199a-5p with *PDPN* were confirmed. RIF patients and the matched control were recruited for endometrium collection. No significant differences were observed between the two groups in endometrial stages, age, and body mass index (*p* < 0.05). The expressions of candidate miRNAs and their proposed targets were examined in the collected endometrium. As shown in Figures 9A,B, *PDPN* and hsa-miR-4306 were downregulated in the endometrial cells of RIF patients compared to that in non-RIF individuals (*p* < 0.05), whereas *PAX2* and hsa-miR-199a-5p were upregulated in the endometrium samples of RIF (*p* < 0.05). These results were consistent with that found from the microarray data. Moreover, we found a negative expression correlation between hsa-miR-4306 and *PAX2* as well as between hsa-miR-199a-5p and *PDPN*, consistent with an inhibitory role of miRNAs on their targets.

To validate the direct targeting relationship between the suggested miRNAs and mRNA, we performed a dual-luciferase assay. Both wild-type and mutated 3′UTR (containing the predicted target sites) luciferase reporter plasmids for *PDPN and PAX2* were constructed. The results showed that overexpression of hsa-miR-199a-5p and hsa-miR-4306 could significantly inhibit the relative luciferase activity of *PDPN-WT3′UTR and PAX2*-WT3′UTR (*p* = 0.0007 for hsa-miR-199a-5p and *PDPN*; *p* = 0.0177 for hsa-miR-4306 and *PAX2*, respectively), whereas the effects were abolished with the mutated 3′UTRs, validating the direct regulatory role of hsa-miR-199a-5p on *PDPN* expression and hsa-miR-4306 on *PAX2* expression (Figure 9C)*.*

## Discussion

miRNAs are evolutionarily conserved small non-coding RNAs that control post-transcriptional gene expression by targeting mRNAs for degradation and translational repression (Lim et al., 2005; Olena and Patton, 2010). miRNAs exert their activity by binding to the 3′-UTR of the target mRNAs, and a single miRNA can regulate multiple target mRNAs simultaneously (Santamaria and Taylor, 2014). Increasing evidence suggests that miRNAs play a key role in the regulation of a range of fundamental cellular processes; thus, abnormal regulation of miRNAs is associated with diseases, including RIF (Catalanotto et al., 2016; Liang et al., 2017; Tan et al., 2020).

Herein, our study initially identified 49 DEMs from the miRNA dataset and 325 DEGs from the mRNA expression profiling dataset, consisting of 42 upregulated and seven downregulated miRNAs, as well as 200 upregulated genes and 125 downregulated genes in RIF sample tissues, compared with healthy endometrial tissues. Enrichment analyses were conducted among these DEMs and DEGs, indicating that these genes were related to the functions of vasoconstriction, regulation of blood vessel diameter, and regulation of systemic arterial blood pressure and to the pathways such as the cell adhesion molecular (CAM) pathway. In addition, a recent study showed a significant reduction in the secretion of mid-term endometrial CAMs in women with RIF, which may be responsible for embryo implantation failure (Guo et al., 2018).

To prioritize the strong candidates for RIF in DEGs, we utilized network analysis which considers gene–gene interactions and can identify central players by the connection degree of each gene. Then, 33 top hub genes with high degrees of three network topology analyses were identified by PPI including 11 upregulated genes and 22 downregulated genes; some of these have previously been reported to be related to RIF. The prostaglandin production by cyclooxygenase 2 (COX2, also known as PTGS2) plays an important role in endometrial receptivity and embryo implantation (Cha et al., 2012). *KDR* (also known as *VEGFR2*) is a hub gene in the PPI network and is overexpressed in RIF compared to fertile samples. Liu et al. (2015) used ultrasound molecular imaging to examine the expression of *VEGFR2* on the uterine vascular endothelium for evaluation of endometrial receptivity. Furthermore, the hub module was significantly enriched in the renin-angiotensin system (RAS)-associated genes such as *AGT*, *AVP*, and *AVPR1A.* In a previous study, AVP receptors were predominantly expressed in non-pregnant women (Fuchs et al., 1998), which is consistent with our finding that *AVPR1A* was upregulated in RIF samples, whereas Pringle *et al.* found that dysregulation of endometrial RAS could be a predisposing factor to endometrial cancer (Pringle et al., 2016). Specifically, they examined the prevalence of RAS-related single-nucleotide polymorphisms and showed that *AGT* levels were less prevalent in women with endometrial cancer than in controls, and *AGT* caused the removal of angiotensin I; both are anti-angiogenic factors. Therefore, an investigation should be made to determine if repression of RAS in the endometrium would influence angiogenesis during embryo implantation and lead to RIF.

miRNA can regulate mRNA expression by direct binding, and most of the time, they have inverse expression correlation. Based on the predicted miRNA (DEMs)–target (DEGs) regulatory network, we found four downregulated and 19 upregulated key miRNAs in the network. Among these, hsa-miR-1260b is known to mediate growth differentiation factor 11 (GDF11)-Smad-dependent signaling, an important regulatory mechanism for the proliferation of vascular smooth muscle cells (VSMCs), which is regulated by hypoxia (Seong and Kang, 2020). In addition, in the regulatory networks, hsa-miR-135b-5p regulated two key downregulated genes: *PDPN* and *AGT*. Overexpression of *PDPN*, a small mucin-like type-1 transmembrane protein and a specific marker for lymph vessel endothelial cells (Quintanilla et al., 2019), significantly increased endothelial cell adhesion, migration, and tube formation, whereas inhibition of *PDPN* expression decreased dermal lymphatic endothelial cell adhesion (Bieniasz-Krzywiec et al., 2019). *PDPN* is a specific marker for the lymphatic endothelium in histopathology (Hamada et al., 2020). Hsa-miR-135b-5p in serum exosome promotes proliferation and migration of *VSMCs* (Zhang et al., 2020) and could be a prognostic biomarker in breast cancer and lupus nephritis (Bao et al., 2019; Garcia-Vives et al., 2020). Suppression of hsa-miR-135b-5p promotes the decidualization of the endometrial stroma and ameliorates the decidualization of human endometrial stromal cells (hESCs) from RIF patients (Wang et al., 2021). Interestingly, has-miR-135b-5p was significantly upregulated in the RIF samples in our study, indicating a suppressed decidualization which finally leads to the RIF. The Has-miR-135b-5p regulatory mechanism in hESC decidualization involved the regulation of transcription factor 3 (*AFT3*) which can promote embryo adhesion *in vitro* by transcriptionally increasing leukemia inhibitory factor expression in epithelial cells (Cheng et al., 2017); in addition, the knockdown of *AFT3* significantly disrupts structural features of decidualized stromal cells (Wang F. et al., 2020). Previous research has also shown that the placental growth factor promotes trophoblast cell invasion by upregulating *ATF3* expression and downregulating hsa-miR-199a-5p expression (Mei et al., 2019), which was overexpressed and targeted on *PDPN* in our study. Hsa-miR-199a-5p stimulates ovarian granulosa cell apoptosis in polycystic ovary syndrome, and its downregulation promotes ovarian granulosa cell viability and inhibits apoptosis, while inducing an increase in serum estradiol (E2) levels and a decrease in serum anti-mullerian hormone levels, luteinizing hormone levels, and follicle-stimulating hormone levels in a polycystic ovarian syndrome rat model (Shao et al., 2020). Moreover, hsa-miR-199a-5p is suggested to play important roles in many cancers such as ovarian cancer, non-small cell lung cancer, and laryngeal cancer in humans (Hua et al., 2019; Gan et al., 2020; Li et al., 2020). Another miRNA that targets *PAX2* is hsa-miR-4306. It is a new therapeutic target for triple-negative breast cancer, which is transcriptionally regulated by estrogen receptor-alpha, human epidermal growth factor receptor 2, and the progesterone receptor (Zhao et al., 2019). In addition, the intercellular transfer of hsa-miR-4306 by platelet microparticles inhibits the migration of human monocyte-derived macrophages through the VEGFA/ERK1/2/NF-κB signaling pathway, which may influence the immunological environment at the maternal–fetal interface (Yang et al., 2019). In addition, its target gene *PAX2* acts as a tumor suppressor in endometrial carcinogenesis (Raffone et al., 2019), which is an accurate marker of precancerous endometrial hyperplasia (Rewcastle et al., 2018). The reduction of PAX2 expression in murine oviductal cells enhances estrogen receptor signaling (Colina et al., 2020).

At this stage, we prioritized 16 key genes and hypothesized that their factors contributing to RIF should be present during the WOI which is critical for embryo implantation. Therefore, we found genes *(PDPN*, *GJA1*, *LPAR3*, and *PAX2*) with significant expression changes during WOI from the single-cell data set of the normal endometrium. The final emphasis placed on *PDPN* and *PAX2* is a result of literature searching and biological interpretation. However, we did not find relevant literature on the role of GJA1 and LPAR3 expression in the endometrium. Then, we choose the two miRNA–target gene pairs hsa-miR-4306-*PAX2* and hsa-miR-199a-5p-*PDPN* for further experimental validation. In addition, we verified their different expressions in the endometrium between RIF patients and the fertile control by qPCR and their target relationship by dual-luciferase assay. These potential regulatory patterns may contribute to the discovery of new molecular targets for the diagnosis of RIF.

There are limitations in the present study. First, the sample sizes of the two microarray datasets aforementioned are small, especially for the miRNA profile dataset. Therefore, future studies with larger sample sizes are required. Second, the results require further investigation and validation both *in vitro* and *in vivo*. Notably, further mechanistic studies of the RIF miRNA–target gene regulatory network could provide insight into the molecular mechanisms of RIF. Furthermore, the detection of these expressions of miRNAs and key genes in the endometrium could be helpful to predict the probability of successful transplantation in clinics. The outcome could help clinicians achieve risk assessment of RIF before IVF treatment and help choose the best treatment strategy during oocyte retrieval and embryo transfer in order to obtain enough embryos available for transfer when faced with RIF and achieve a satisfactory clinical outcome.

## Data Availability

Publicly available datasets were analyzed in this study. These data can be found here: https://www.ncbi.nlm.nih.gov/geo/; (GSE71332, GSE111974, GSE111976).
