# Corrigendum: Identification of key endometrial microRNAs and their target genes associated with pathogenesis of recurrent implantation failure by integrated bioinformatics analysis

**DOI:** 10.3389/fgene.2022.979941

**Published:** 2022-08-10

**Authors:** Jin Shang, Yan-Fei Cheng, Min Li, Hui Wang, Jin-Ning Zhang, Xin-Meng Guo, Dan-dan Cao, Yuan-Qing Yao

**Affiliations:** ^1^ Medical School of Chinese People’s Liberation Army (PLA), Beijing, China; ^2^ Shenzhen Key Laboratory of Fertility Regulation, Reproductive Medicine Center, The University of Hong Kong-Shenzhen Hospital, Shenzhen, China; ^3^ Shenzhen Institute of Advanced Technology, Chinese Academy of Science, Shenzhen, China; ^4^ Department of Obstetrics and Gynecology, The Seventh Medical Center, Chinese PLA General Hospital, Beijing, China; ^5^ Department of Obstetrics and Gynecology, The First Medical Center, Chinese PLA General Hospital, Beijing, China; ^6^ College of Medicine, Nankai University, Tianjin, China

**Keywords:** recurrent implantation failure, differentially expressed genes, differentially expressed miRNAs, bioinformatics, endometrial transcriptomics

In the published article, there was an error in author Dan-dan Cao’s listed affiliation. Instead of “Dan-dan Cao^3*^,” it should be “Dan-dan Cao^2*^.”

The authors apologize for this error and state that this does not change the scientific conclusions of the article in any way. The original article has been updated.

